# Long-Term Doctor-Patient Relationships: Patient Perspective From Online Reviews

**DOI:** 10.2196/jmir.2552

**Published:** 2013-07-02

**Authors:** Alissa Detz, Andrea López, Urmimala Sarkar

**Affiliations:** ^1^UCLA Division of General Internal Medicine and Health Services ResearchUniversity of California, Los AngelesLos Angeles, CAUnited States; ^2^Center for Vulnerable PopulationsDivision of General Internal MedicineUniversity of California, San FranciscoSan Francisco, CAUnited States

**Keywords:** social media, qualitative, primary care

## Abstract

**Background:**

Continuity of patient care is one of the cornerstones of primary care.

**Objective:**

To examine publicly available, Internet-based reviews of adult primary care physicians, specifically written by patients who report long-term relationships with their physicians.

**Methods:**

This substudy was nested within a larger qualitative content analysis of online physician ratings. We focused on reviews reflecting an established patient-physician relationship, that is, those seeing their physicians for at least 1 year.

**Results:**

Of the 712 Internet reviews of primary care physicians, 93 reviews (13.1%) were from patients that self-identified as having a long-term relationship with their physician, 11 reviews (1.5%) commented on a first-time visit to a physician, and the remainder of reviews (85.4%) did not specify the amount of time with their physician. Analysis revealed six overarching domains: (1) personality traits or descriptors of the physician, (2) technical competence, (3) communication, (4) access to physician, (5) office staff/environment, and (6) coordination of care.

**Conclusions:**

Our analysis shows that patients who have been with their physician for at least 1 year write positive reviews on public websites and focus on physician attributes.

## Introduction

In the United States, recent health reform legislation has increasingly emphasized patient-centered care and patient satisfaction within primary care. Patients often have a choice when selecting a primary care physician. Therefore, patient reviews of their experiences may influence choice of physician as well as physician practices.

Continuity of patient care is one of the cornerstones of primary care [1]. Previous research indicates that both patients and physicians value this aspect of outpatient medical care [2-4]. Moreover, continuity of care is associated with improved management of chronic disease, increased administration of preventative health services, and fewer emergency department visits and hospitalizations [5-11].

The patient-centered medical home is a model of providing primary care defined by management of a population of patients rather than provision of care during periodic primary care visits. This model emphasizes patient-centeredness, accessibility, and comprehensive and coordinated care with a focus on patient safety and quality. The medical home model has been increasingly promoted as a means to improve primary care in the United States and emphasizes continuity between patient and provider as a core component [12-14].

Despite the importance of continuity, there are few studies dedicated to defining what factors are important for establishing and maintaining a relationship with a given physician over time. Since promoting continuity of care is an explicit goal in providing quality primary care, identifying factors that promote continuity is critical. In turn, this requires understanding patient perspectives on long-term relationships with primary care physicians.

Although Internet website reviews of physicians are controversial [15-17], they do provide unfiltered data regarding patient perceptions of health care. These reviews can complement existing studies on the patient-physician relationship. Traditional structured satisfaction surveys have been shown to perform differently across patient populations and may not capture the views of all patients [18-21]. Public websites allow individuals to review their physicians in an anonymous and unstructured format. Evaluating these publically available unstructured reviews may give us additional insight into what factors of the patient-doctor relationship are particularly important to patients. In this study, we examine publicly available, Internet-based reviews of adult primary care physicians, specifically written by patients who report long-term relationships with their physicians. We employed qualitative analysis to undercover themes within reviews of long-term patient-physician relationships.

## Methods

### Design

This substudy was nested within a larger qualitative content analysis of online physician ratings. The methods of the parent study are described in detail elsewhere [22]. The parent study was a qualitative content analysis of 712 online reviews from two publicly available rating websites (Yelp, a general rating site and RateMDs, a physician-rating website). For the parent study, we purposively sampled reviews of 445 primary care doctors (internists and family practitioners) from four geographically dispersed urban locations in the United States.

For this substudy, we focused on reviews reflecting an established patient-physician relationship. We chose this subset of reviews due to our interest in continuity of care. We defined long-term patients as those seeing their physicians for at least 1 year. There is a lack of consensus about what constitutes a long-term patient-physician relationship. Time frames are commonly defined by either number of visits or calendar time. We elected to use a 1-year time frame because previous investigators [23-26] and multiple Internet reviews in our dataset referenced this time frame.

### Sampling

**Figure 1:**
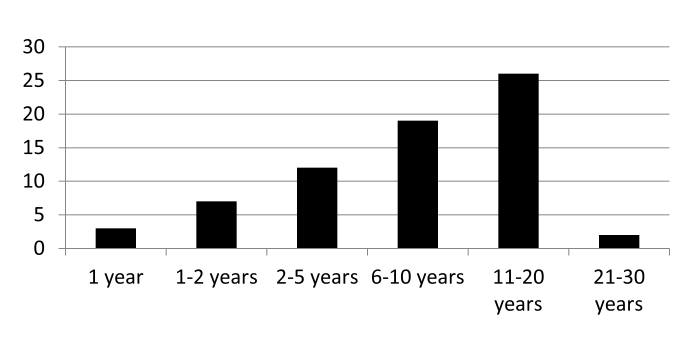
Years with primary care physician.

In the parent study, our search strategy was meant to mimic two popular ways of searching for ratings using the Internet: (1) using a search engine and (2) using a well-known general ratings site. First, to mimic a patient’s approach, we utilized the popular Google search engine. When we entered the phrase “rate doctor” into Google.com, the first result was for the website RateMDs. As its name suggests, RateMDs exclusively rates physicians. Second, because we surmised that patients might search for physician ratings on a website they use for other types of consumer ratings, we selected the website Yelp. Our sampling strategy had two distinct levels because each physician could have multiple reviews. Each website first generates a list of physicians. Because the order in which doctors were listed on the website is nonrandom, we prespecified our sampling of physicians as follows: We selected 30 reviews of doctors appearing at the beginning of the search results list, 40 reviews of doctors appearing in the middle of the search results list, and 30 reviews of doctors appearing at the end of the search results list. Next, we purposively sampled the first three available reviews for each individual physician. We analyzed reviews that patients posted publicly. We de-identified physicians (the reviews’ subjects) and identified overarching themes rather than focusing on individual performance. Moreover, the patients (review authors), who knowingly posted reviews publicly, did so with varying degrees of anonymity (true name vs Yelp username) and revealed differing amounts of personal data. For ethical reasons, we chose to de-identify review author data prior to analysis, even for individuals who designated their information as public. Utilizing the parent study, we extracted all patient reviews that referred to amount of time with their physician. Of 712 reviews, 3 patients specified a relationship with their physician of 1 year, 7 specified a relationship of 1-2 years, 74 specified a relationship of greater than 2 years, and 16 patients did not specify a number of years but implied a long-term relationship through their comments (“several years”). The remainder of reviewers did not specify length of time with a physician (Figure 1).

### Qualitative Analysis

As explained in the parent study, we developed preliminary codes of all reviews by applying content analysis theory to a sample set of 50 reviews [27,28]. When developing our codes, we incorporated themes from the literature about factors in patient-physician encounters that impact patient satisfaction [29,30].

Two investigators independently coded 328 (46%) of the reviews, and the remainder of the reviews were coded by 1 investigator. Codes were created as new themes emerged and thematic saturation was achieved after 100 reviews. A total of 60 codes were used for all reviews. All analyses were performed using Atlas.ti software.

In this study, we focus on the themes and global domains found in reviews by patients who have been with their physician for at least 1 year. We describe general characteristics of Internet reviews by long-term patients and compare their comments to other reviews of primary care physicians.

## Results

Of the 712 Internet reviews of primary care physicians, a total of 93 reviews (13.1%) were from patients that self-identified as having a long-term relationship with their physician, eleven reviews (1.5%) commented on a first-time visit to a physician, and the remainder of reviews (85.4%) did not specify the amount of time with their physician. Of the reviews by long-term patients, 39% were from Yelp and 57% were from RateMDs. Long-term patients were more likely to reflect positively about their physician (86%). In contrast, only 55% of the other patients wrote positive reviews.

**Figure 2:**
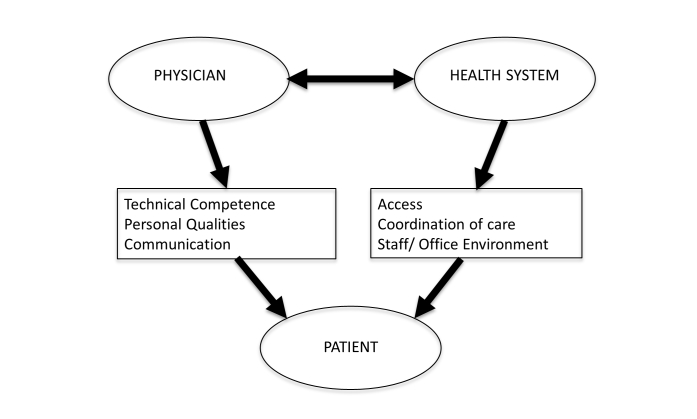
Conceptual model.

Analysis of long-term patient reviews also revealed six overarching domains: (1) personality traits or descriptors of the physician, (2) technical competence, (3) communication, (4) access to physician, (5) office staff/environment, and (6) coordination of care (see Figure 2). The first three domains relate directly to qualities of an individual physician while the subsequent domains reflect the physician practice and health care system (see Table 1). Overall, the reviews by long-term patients emphasized physician individual attributes. The three most prevalent themes were (1) empathy, eg, “My doctor is caring” and (2) overall excellence, eg, “Dr. X is the best”, both of which fell in the domain of personality traits/descriptor, and (3) fund of knowledge, eg, “Dr. X is very knowledgeable”, which is an aspect of technical competence.

### Personality Traits

Most reviews by long-term patients discussed one or more physician qualities; 92% of descriptors mentioned by long-term patients were positive, and the most common themes were “amazing” and “empathetic”. Other qualities frequently mentioned by long-term patients included “helpful”, “professional”, “calm”, and “detailed”. While some reviews included specific examples, many simply included a positive descriptor. Reviews by patients with either short-term or unspecified relationships with a physician also commonly included physician descriptors, but comments were more likely to be negative (18% versus 8%). Negative descriptors included “antagonistic”, “rushed”, and “condescending”.

### Technical Competence

Physician competence was highlighted in 41% of reviews by long-term patients. These reviews discussed knowledge or clinical decision making of the physician. The patients included anecdotes describing accurate and prompt diagnosis. One reviewer remarked, “She detected my medical problem when others had missed it”. Virtually all of the comments in this domain (92%) were positive.

### Communication

Communication skills of the physician during a clinical encounter were described in 34% of the comments by long-term patients and 22% of all other reviews. Comments about this domain focused on physician listening skills, eg, one review stated, “[the physician] always listens to what I have to say”. Other reviews referenced the ability of a physician to explain a diagnosis or new medication. One patient who remarked, “[He] explains the meds that he prescribes, he listens to and answers my questions”. Notably, regardless of the length of the relationship, comments about communication were favorable (94% for long-term patient and others).

### Access to Physician

We defined access as the ability to make an appointment, contact a physician, or be seen in a timely manner during a clinic visit. Descriptions of wait time and experience making an appointment were included in this domain. Many comments focused on this domain, and they were more varied than comments regarding individual attributes. While some positive comments described physicians as “accessible”, negative comments about difficulty making an appointment or excessive wait times at the office were noted, even among patients with an established relationship with their physician. Reviews by long-term patients were generally favorable about experiences making an appointment but unfavorable regarding time waiting for a scheduled appointment. One patient complained, “[I] waited almost 2 hours even though I had an appointment!” In fact, wait time was the only theme where negative comments outweighed positive comments in long-term patient reviews.

### Office Staff/Environment

**Figure 3:**
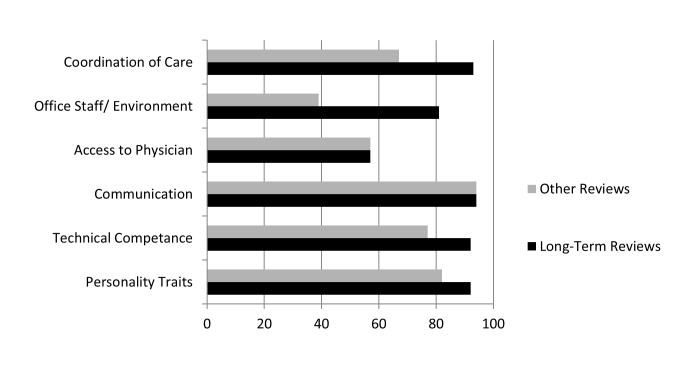
A comparison of long-term reviews and other reviews.

This domain includes all aspects of the medical visit apart from the face-to-face patient-physician interaction. Comments often referenced personality traits and helpfulness of the office staff. As an example, one patient wrote, “Staff is great—friendly and quick to respond”. In this domain, there were clear differences between long-term patient reviews and other reviews, as long-term patients tended to comment favorably on nonphysician office staff (72%), while non–long-term patients complained about staff more often than giving them positive reviews. Moreover, long-term patients were less likely than other reviewers to include descriptions of the office environment (see Figure 3).

### Coordination of Care

We incorporated referrals and any communication between a physician and patient outside of the individual office visit under the domain of coordination of care. Only 16% of comments by long-term patients referred to this aspect of their care, and as for physician attributes, virtually all reflections were positive. Patients described receiving prompt communication with their physician regarding laboratory test results and being pleased with the referrals their primary care physician arranged. For example, one long-term patient wrote, “When necessary, he refers me to other excellent doctors and specialists”. In contrast, patients with short or unspecified relationships with a physician expressed dissatisfaction with coordination of care, as exemplified by one review that expressed, “He offered no guidance on referrals, sent me to a horrible GI”. Table 2 shows the results for these three domains.

**Table 1:** Major themes in reviews of long-term patients.

Domain	Themes	Example Quote	Number of comments^a^	Positive comments (%)	Negative comments (%)
**Personality traits**					
	Empathy Overall excellence	Shows concern and competence as well as being kind, warm and friendly and respectful. I know he really cares He’s a great doctor and a great person.	118	92	8
**Technical competence**					
	Knowledge Decision making	The guy knows his stuff - his diagnoses have always been decisive & spot on She has an excellent knowledge of medicine	38	92	8
**Communication**					
	Listening Explaining	I feel I can tell him or ask him ANYTHING, which is vital with your personal physician. Dr. X spends time listening to what's going on in my life and asking good questions about my health. He is a doctor who listens and talks to you like a person and not an object. He is also willing to answer any question you have and explain it in a way that a lay person can understand.	32	94	6

^a^For the number of comments, we included each instance the domain was referenced within a review. For some reviews, a domain could be mentioned more than once.

**Table 2:** Themes in patients’ reviews.

Domain	Themes	Example Quote	Number of comments^a^	Positive comments (%)	Negative comments (%)
**Access to physician**					
	Making an appointment Wait time	Wait times in the office in general can be VERY long (I once waited 2 hours). Dr. X is always able to squeeze me in last minute when I am feeling sick. Night or day, he is available.	37	57	43
**Staff/ office environment**					
	Staff Office environment	Her office staff is great, always getting me in for an appointment after they realize what a huge worrier I am. Staff is great—friendly and quick to respond His office looks like an art gallery.	26	81	19
**Coordination of care**					
	Follow-up Referral—communication of test results	He called me personally with my results even though they were all normal. When necessary he refers me to other excellent doctors and specialists.	15	93	7

^a^For the number of comments, we included each instance the domain was referenced within a review. For some reviews, a domain could be mentioned more than once.

## Discussion

### Principal Results

Achieving continuity is important for providing quality primary care, and understanding factors patients perceive as important to long-term patient-physician relationships provides insight into promoting this continuity. Internet reviews, while limited, offer a novel perspective that can add to findings from more traditional patient satisfaction assessments. Existing patient satisfaction surveys regarding perceptions of individual primary care physicians are limited by low response rates and underrepresenting patients who are younger, poorer, less well educated, and not white [18-21]. Responders to these surveys tend to express higher satisfaction than nonresponders creating bias and overestimating patient satisfaction [31]. Our study provides unique insight into the patient’s view of the patient-physician relationship and aspects that foster continuity.

Our analysis shows that patients who have been with their physician for at least 1 year write positive reviews on public websites and focus on physician attributes. Comments by established patients were more positive than other reviews, both regarding physician characteristics and technical competency. It is not surprising that patients who have been with a physician for at least 1 year write positive comments on Internet rating sites. This is consistent with previous research demonstrating an association between patient satisfaction and continuity of care [32-35]. A patient that is satisfied with encounters is more likely to return and see a given physician. Moreover, the sustained relationship likely enhances satisfaction by promoting trust and an interpersonal connection.

Personal characteristics were included in most reviews by long-term patients with positive descriptions of their physician. The positive comments about physicians’ individual characteristics are consistent with other sources for evaluating patient satisfaction. This shows not only that the importance of an interpersonal connection for establishing and maintaining continuity [35-37], but also that Internet reviews reflect some similar patient values to traditional methods for measuring patient satisfaction.

The most common themes of empathy, overall excellence, and knowledge reflect aspects of medical care that promote continuity for these patients. Prior studies of patient perceptions of primary care physicians have also demonstrated the value of these factors. Empathy and patient-centered care have been associated with patient satisfaction and improved clinical outcomes [38-41]. Thus, the fact that Internet reviews also capture these factors suggest that they merit further study. Patient satisfaction is also influenced by perceived technical skill of a physician [37,42].

Factors beyond the face-to-face physician interaction also surfaced in Internet reviews. Specifically, long-term patients commented favorably on staff and office environment. While not directly influencing medical decision making, the office environment and staff may impact a patient’s impression during a clinical visit. Moreover, office staff are a part of the medical team that can facilitate or impede appropriate care. The relationship between negative perceptions of staff and patient continuity and follow-up should be specifically addressed. It is notable that long-term patients commented about staff less frequently, and it is possible that the influence of nonphysician factors wanes with duration of patient-physician relationships.

In addition, access was the most commonly included nonphysician factor in reviews by long-term patients. Previous research demonstrates that being seen within a day and having a short wait time correlates with improved patient satisfaction [35,43]. The Internet reviews show a similar emphasis, highlighting that the ability to make an appointment and be seen in a timely manner are important to patients. Time waiting in the waiting room for a given appointment was the only factor that caused dissatisfaction, regardless of the number of visits to a physician. The analysis of reviews from established patients indicate that patients are willing to tolerated suboptimal waiting times for physicians in whom they have trust and confidence. This is exemplified by one reviewer who stated, “This results in us having to wait for our appointment to be taken, but once taken, we know that she’ll do a good job of helping us”.

### Limitations

Despite this being the first study, to our knowledge, to use public Internet-derived data to gain insight into factors associated with long-term patient-physician relationships in primary care, our findings are consistent with studies examining patient perspectives online in the context of specific health conditions like diabetes [44].

We acknowledge that our study has several limitations. First, as with all analyses using nonstandardized data, we cannot comment on the broader prevalence of the themes we uncovered in our sample. Second, as with all patient satisfaction studies, the self-selection of patients writing reviews on public websites introduces bias and may limit the generalizability of our findings. Of note, a different subset of patients are likely to complete Internet reviews than those that complete traditional patient satisfaction surveys [19,20]. Therefore, our findings may capture a novel patient perspective. Third, the majority of patients writing Internet reviews did not report the length of time with their physician. Thus, we were unlikely to have captured all patients that were truly with their physician for longer than 1 year [32].

Despite these limitations, our findings contribute to existing knowledge regarding the patient perspective of primary care. In particular, our data show the factors important in establishing and maintaining a relationship with a physician over time.

### Conclusions

Our research also adds to the data regarding public websites that enable patients to review individual physicians. Patient use of the Internet regarding health care has dramatically increased with 80% of American Internet users looking online for health information and 16% viewing reviews of health care providers [45]. The use of such websites has generated controversy both in the media and in the medical literature [15,46,47]. Research regarding the content of these websites has just begun to emerge [48-50]. Our results suggest that concerns about Internet rating affecting one’s professional reputation may be overstated, as the majority of patient reviews were positive.

Website reviews of physicians are a reality and could serve as an important tool for patients as well as health care providers. Our analysis suggests ways that websites could be restructured to provide more easily accessible and reliable data. For example, differences clearly exist between individuals who have had a few visits to a physician and those with a well-established relationship with their provider. This suggests that length of time with a physician should be specified when patients write reviews. In addition, our analysis suggests that common themes emerge in reviews. Standardization of websites to direct content of reviews may also make sites more helpful to guide consumers and to guide changes in primary care practices. For physicians, reviews can provide insight into behaviors and attitudes that keep their patients engaged with care over time and also provide needed information about aspects of the visit beyond the physician-patient encounter. Factors such as staff complaints and ease of appointment-making may not be apparent to physicians but could be improved if patients’ concerns were known. Further research is needed to track development of these websites, to validate structuring of website reviews, and to study how reviews impact physician practice and patient choice of their physician.
